# Carpal tunnel syndrome and the use of computer mouse and keyboard: A systematic review

**DOI:** 10.1186/1471-2474-9-134

**Published:** 2008-10-06

**Authors:** Jane F Thomsen, Fred Gerr, Isam Atroshi

**Affiliations:** 1 Department of Occupational Medicine, Copenhagen University Hospital in Glostrup, Nordre Ringvej, DK-2600 Glostrup. Denmark; 2 Department of Occupational and Environmental Health, College of Public Health, University of Iowa, Iowa City, IA 52245. USA; 3 Department of Orthopaedics, Hässleholm and Kristianstad Hospitals SE-28125 Hässleholm. Sweden

## Abstract

**Background:**

This review examines evidence for an association between computer work and carpal tunnel syndrome (CTS).

**Methods:**

A systematic review of studies of computer work and CTS was performed. Supplementary, longitudinal studies of low force, repetitive work and CTS, and studies of possible pathophysiological mechanisms were evaluated.

**Results:**

Eight epidemiological studies of the association between computer work and CTS were identified. All eight studies had one or more limitation including imprecise exposure and outcome assessment, low statistical power or potentially serious biases. In three of the studies an exposure-response association was observed but because of possible misclassification no firm conclusions could be drawn. Three of the studies found risks below 1. Also longitudinal studies of repetitive low-force non-computer work (n = 3) were reviewed but these studies did not add evidence to an association. Measurements of carpal tunnel pressure (CTP) under conditions typically observed among computer users showed pressure values below levels considered harmful. However, during actual mouse use one study showed an increase of CTP to potentially harmful levels. The long term effects of prolonged or repeatedly increased pressures at these levels are not known, however.

**Conclusion:**

There is insufficient epidemiological evidence that computer work causes CTS.

## Background

Carpal tunnel syndrome (CTS) is a compression neuropathy of the median nerve as it passes through the carpal tunnel. It is regarded as the most frequent compression neuropathy. Based on both clinical symptoms and nerve conduction tests (NCT), overall prevalences of 3.0–5.8% among women and 0.6–2.1% among men have been found in general population samples [1,2]. CTS is generally believed to be caused by increased pressure in the carpal tunnel. It has been a matter of discussion whether biomechanical factors may cause the condition. It is now widely accepted that exposure to hand-arm vibrations and exposure to a combination of repetitive hand use and the use of hand force may be causal agents [3]. In recent years, with the expanding use of computers, it has been a matter of concern if computer use could be a risk factor for the development of CTS, and if so, should the condition be recognised as an occupational disease. The issue was partly addressed in a recent review where it was concluded that the evidence did not point at any important association between keyboard and computer work and CTS [3]. The review, however, did not include a systematic search for studies on computer work and the evaluation was based on a limited number of epidemiological studies only. Furthermore, there were no considerations on possible mechanisms.

Thus, the core of this review was a detailed evaluation of the existing epidemiological evidence of an association between computer work and CTS. Because of some similarities between computer work and repetitive, low force work, studies examining associations between repetitive work and CTS were also evaluated (longitudinal studies only). Though not epidemiological in design, studies of median nerve function among computer users, exposure characteristics in computer work and the influence on carpal tunnel pressure (CTP) and median nerve function was also evaluated in order to determine possible pathophysiological mechanisms.

The review was originally conducted on behalf of the Scientific Committee of the Danish Society for Occupational and Environmental Medicine for use by The Danish National Board of Industrial Injuries in its evaluation of whether specific musculoskeletal disorders among computer workers should be included on its list of occupational injuries and diseases that may be compensated through the Danish Workers' Compensation Act [4].

## Methods

### Literature search

The identification of epidemiological studies examining associations between computer work and CTS began with a search in the following databases: Pubmed, Embase, Web of Science, and Arbline. The language had to be English and the article published in a journal with a peer-review process. Only papers with original data were considered. The original search was performed in June 2005 and covered all years included in the databases. For the purpose of the present review this part of the search was further updated in August 2008 (human studies only).

The text search terms were: 'carpal tunnel syndrome or CTS or median nerve and computer or visual display unit or keyboard or mouse'. All titles and relevant abstracts were read. Reference lists in relevant articles and personal files were also searched for articles not identified in the original database search.

The criteria for inclusion in the review of epidemiological evidence were: Cross-sectional or longitudinal studies that included participants exposed to computer work (mouse or keyboard) or typing and participants without such exposure and case-referent studies where computer work (mouse or keyboard) or typing was specified as an exposure. In all studies, the health outcome had to be CTS ascertained with 1) symptoms (questionnaire or interview or both) in combination with NCT or 2) symptoms alone but confirmed by qualitative interview.

Studies in which CTS was ascertained with questionnaire symptoms only, NCT only or Tinel's or Phalen's test only were not included. Studies using workers' compensation data were not included (Table 1).

**Table 1:** Inclusion and exclusion criteria for the epidemiological studies of the association between computer work and carpal tunnel syndrome.

CTS: Carpal tunnel syndrome
NCT: Nerve conduction test
**Inclusion criteria**

English language, peer-reviewed articles with original data
Study design
Longitudinal studies
Cross sectional studies
Case referent studies
Population
Should include both an exposed group and a control group
Exposure
Computer work (keyboard or mouse) or typing should be defined as the exposure
Outcome definition
1. Symptoms (questionnaire or interview or both) of CTS in combination with NCT or 2) Symptoms alone but confirmed by qualitative interview.

**Exclusion criteria**

Population
Studies using workers' compensation data
Outcome definition
Studies where CTS was diagnosed using questionnaire symptoms only, NCT only, or clinical tests as Tinel's and Phalen's tests only

Supplementary to the epidemiological evidence of the risk of computer work, longitudinal studies of the association between CTS and high repetition and low force work were identified with the search terms 'carpal tunnel syndrome and repet*' (Pubmed only). All titles and relevant abstracts were read. Only studies with a prospective design and a focus on the association between repetitive work and CTS as defined above were included.

With the use of the above search terms studies of median nerve function in computer users were retrieved and included. The search term 'computer use and ergonomic risk factors' was also used (Pubmed only). Studies describing the arm and hand position and force level in computer work were retrieved and included.

Finally, the search term 'carpal tunnel pressure' was used (Pubmed only). All titles and relevant abstracts were read and human studies with a focus on carpal tunnel pressure (CTP) and the effect of force and position of arm, wrist and fingers were included. No cadaver studies were considered.

In the quality assessment of each of the epidemiological studies, no scoring system was used. Instead, descriptive data for each study were provided and assessed, as follows: Study design: Longitudinal better than cross sectional and case referent but dependent on other of the mentioned factors as well. Exposure assessment: Objective measurements better than self-reported exposure better than job title. Outcome definition: Symptoms and NCT better than symptoms only. Length of follow-up period: If baseline exposure was used then a short follow-up period was better than a long period. Controlling for potential confounders: Age and gender as a minimum. Reporting or selection bias was considered. Sample size was assessed in relation to statistical power. Blinding of the participant and/or the examiner was considered.

### Level of evidence

The overall evaluation was based on a classification system established by The Scientific Committee of the Danish Society of Occupational and Environmental Medicine, 2005 and used in other recent reviews, e.g. [3,5]. The following categories were used:

Strong evidence of a causal association: A causal relationship is very likely (chance, bias, and confounding could be ruled out with reasonable confidence) between an exposure to a specific risk factor and a specific outcome. A positive relationship has been observed between exposure to the risk factor and the outcome in several studies.

Moderate evidence of a causal association: Some convincing epidemiological evidence exists (chance, bias, and confounding are not the likely explanation) for a causal relationship between an exposure and a specific outcome. A positive relationship has been observed between exposure to the risk factor and the outcome in several studies.

Limited evidence of a causal association: Some convincing epidemiological evidence exists in some studies for a causal relationship between an exposure to a specific risk factor and a specific outcome. A positive relationship has been observed between exposure to the risk factor and the outcome, but it is not unlikely that this relationship could be explained by chance, bias, or confounding.

Insufficient evidence of a causal association: The available studies are of insufficient quality, consistency, or statistical power to permit a conclusion regarding the presence or absence of a causal association.

The likelihood that chance, bias and confounding may explain observed associations was based on the quality assessment criteria (study design, exposure assessment, outcome definition, confounder control, length of follow-up period, selection and information bias, study size and statistical power).

Biological plausibility and contributory information may add to the evidence of a causal association.

## Results

### Epidemiological studies

#### Computer work and carpal tunnel syndrome

**Figure 1:**
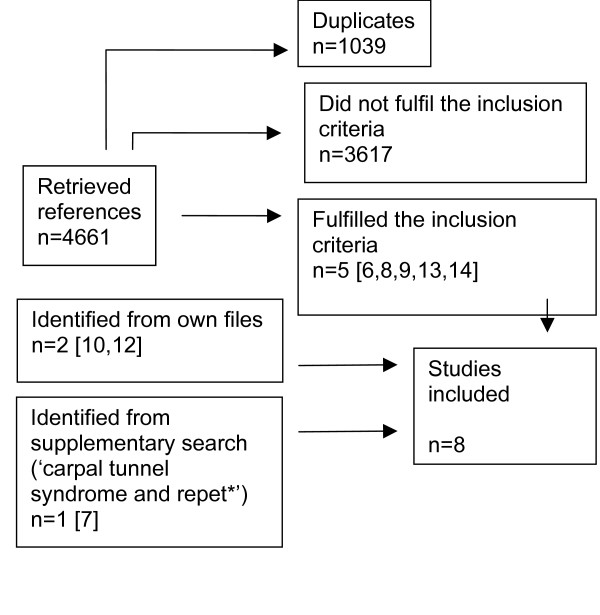
Flow-chart showing the identification of epidemiological studies of the association between computer work (keyboard and mouse) or typing and carpal tunnel syndrome.

In the literature search on the association between computer work and CTS 4661 references were identified (Figure 1). Eight epidemiological studies met the criteria for inclusion [6-14] (two of the papers were from the same population [11,12]). Four of these studies were prospective in design [6-8,12], one was a case-referent study [10], one was cross-sectional but with a case-referent approach [9], and two were cross-sectional[13,14]. The studies are listed in Table 2 with information on design, population, response rate, control group, exposure, CTS case definition, confounders controlled for, results and strengths and weaknesses [see Additional file 1].

The large population-based study by Atroshi et al. included both physical examination and NCT [14]. It showed a significant *protective *effect of keyboard work, i.e. the prevalence of CTS increased with decreasing hours/day with self-reported computer work. The study was carefully conducted with adequate blinding of the interviewer regarding exposure and of the technician regarding the symptom status.

An Indian cross-sectional study, on the contrary, showed a significant effect of both years and hours/day with computer work [13]. The participation rate was 100% which raises a question of the selection process. There was not the expected effect of gender (men had twice the frequency of CTS compared to women), age or BMI. Blinding was not described and NCT was not used.

In the follow-up study of Andersen et al. (The NUDATA study), two case definitions relevant for this review were used [see additional file 1] [8]. When applying case definition 1, analysis of baseline data showed odds ratios of 2.3 (95% CI 1.2–4.5) among participants reporting 5–9 h/w of mouse use increasing to 3.6 (95% CI 1.8–7.1) among participants reporting 20–24 h/w of mouse use. There was no further increase of risk among participants reporting more than 24 h/w of mouse use. Case definition 2 showed only borderline significance at 30+ hours and a very irregular pattern of association. In the follow-up analyses, a significantly increased risk was found among participants reporting working more than 20 h/w with the mouse.

In the extensive prospective study of computer users by Gerr et al. the prevalence of CTS was too low to allow any analyses of association [6]. It is worth noting, however, that with the use of an outcome definition including both symptoms and NCT the prevalence and incidence of the disease was low in this working population.

As a substudy of the Danish PRIM study (Project on Intervention and Research in Monotonous work), 731 participants from three companies, one bank and two postal centres, were included. [7]. Data entry was the main repetitive task. Electrogoniometer measurements of wrist movements showed that this work task was highly repetitive. Blinding of physician and technician was observed. The overall prevalence of CTS was 1.1% (8 cases) on the working hand and 0.3% (2 cases) on the contralateral hand. The risk of CTS was statistically significantly increased for every 10 hours of repetitive work (OR = 1.86 (95% CI 1.06–3.19)) after adjusting for forceful work and personal characteristics. The annual incidence of CTS was 0.62% (4 cases) and thus no further analyses of incidence data could be made.

Nathan et al. had followed a cohort established in 1984 for 11 years [11,12]. Originally, the cohort included 471 participants from 4 industries representing a wide variety of hand activities. It was not stated if the at-risk population had normal NCT at baseline nor if the examiners were blinded to exposure or health status. In multivariate analyses adjusting for various potential baseline confounders (but not age and gender), neither an effect of keyboard work nor of repetitive work (as ascertained in 1984) was found.

In the study by Stevens et al., all participants were identified as "frequent computer users" working in a medical facility [9]. Exposure and demographic characteristics were compared between the two groups but without formal statistical testing. Hours of daily keyboard use and years with keyboard did not differ between the groups. Frequent mouse use was more prevalent in the CTS group than the non-CTS group (48.1% vs. 27.9%). This difference was not tested statistically. However, testing the reported figures with a Mantel-Haenszel chi-square test (1 df) showed a statistically significant difference (p = 0.04).

In another study with a case-referent approach de Krom et al. identified 156 CTS cases from a population based survey and from an outpatient department of neurology [10]. Referents were persons without CTS symptoms from the population based survey (n = 473). Care was taken to keep the purpose of the study blinded to the participants in order to avoid information bias. One question documented hours per week of typing during the last 5 years. The odds ratios were all below 1 and not statistically significant.

A large population based study with main focus on hand-arm vibration and CTS did not meet the inclusion criteria because of insufficient outcome definition [15]. There was one questionnaire item concerning keyboard use more than 4 hours per day. No excess risk for self reported tingling/numbness was found (PR = 1.1, 95% CI 0.8–1.3) after adjusting for age, smoking, headaches and tiredness/stress.

In summary, of the eight studies identified, four studies were designed as follow-up studies. One of these studies was well performed with a short follow-up period, a large study population and thus sufficient statistical power. The study found some positive associations. However, the possibility of information bias in combination with an outcome definition not involving NCT made inferences difficult [8]. Two of the follow-up studies had too few CTS cases to perform analyses [6,7]. The last follow-up study identified had a very long follow-up period, only baseline exposure information and no adjustment for age and gender. They found no association but because of the important limitations the result was difficult to interpret [12]. One of the follow-up studies reported positive associations from baseline data but here the computer exposure was mixed with other kinds of repetitive work [7]. One of the two cross-sectional studies was very well performed and had enough statistical power and found the opposite association of what was expected [14]. The other cross-sectional study showed a positive association but had some confusing results, e.g. a much higher prevalence among men compared to women. Two of the eight studies were case-control studies. One study had limited statistical power and showed no association [10]. The other showed a weak association but only in a crude analysis with no adjustment for age [9].

Repetitive work and carpal tunnel syndrome

In the literature search on repetitive work and CTS, 229 studies were retrieved and three studies fulfilled the inclusion criteria [16-18]. Two of the three studies were based on one population [16,17]. Werner et al. identified 49 asymptomatic participants with an abnormal NCT and 59 with a normal NCT and assessed them after 17 months and after 70 months. Work tasks were video-filmed and categorized according to repetition rate on a scale from 1–10. The incidence of developing CTS symptoms was equal in the two groups after 17 months but significantly higher after 70 months in the group with initially positive NCTs. Repetitive work was a risk factor for developing symptoms after 17 months (OR 1.35, 95% CI 1.03–1.77) and after 70 months (risk estimate not provided).

In a well designed follow-up study by Gell et al. no association between level of repetitive hand tasks and development of CTS was found [18]. The case definition included both symptoms as well as NCT. Each job was assessed and rated for ergonomic exposures.

In all, results from studies on other kinds of repetitive, low force work and CTS did not add to evidence of to an association between computer use and CTS.

#### Studies of nerve involvement among computer workers

Seven studies comparing median nerve function in computer users with groups without computer use were found [19-25]. Two of the studies assessed median nerve function with NCT [23,25] whereas the other five studies used vibration perception threshold testing. Vibration sense perception, however, is not a good indicator of CTS [26]. The selection of participants was not described in the five studies using vibration perception threshold testing and selection bias may have affected the results.

The study of Murata et al[23] used nerve conduction tests among 27 female life insurance company employees entering data for six hours or more per day and 24 female students. Significant differences in median nerve sensory conduction velocities were found for measurements across the carpal tunnel whereas values proximal and distal to the wrist did not differ. The two groups differed in symptom profile. The findings of Murata et al[23] were in contrast to a recent study by Sandén et al[25] in which 82 secretaries with a median of 6 hours of daily computer work were compared to 35 nurses with very limited computer work. No statistically significant differences were found in the median nerve conduction velocity or in the vibration threshold between the two groups in t-test analyses. Doezie et al. compared vibration thresholds among transcriptionists with symptoms to a control group [21]. Thresholds of the second and fifth fingers were significantly elevated in the transcriptionists compared to the control group but only for the high frequencies (125–500 Hz). Very little information about the control group was shown.

Greening et al. conducted two studies examining associations between vibrotactile thresholds and computer use [19,20]. In both studies, they found that patients with musculoskeletal symptoms in the upper limbs had higher thresholds than healthy individuals. The studies did not compare computer users without symptoms to non-computer users. Thus, the design of the studies did not allow conclusions considering an effect of computer work per se.

Similar methods were used in a Danish study and similar results were observed [24]. Finally, another Danish study studied vibration thresholds in computer users but with a focus on symptoms and not levels of computer use [22].

#### Pathophysiological mechanisms

It is widely believed that biomechanical factors (e.g. forceful exertions, repetition, and awkward postures) increase the risk of CTS by increasing carpal canal pressure with subsequent nerve ischemia [27]. Therefore, in addition to epidemiological evidence of associations between computer work and CTS, insight into the role of computers on the development of CTS may be found in studies examining wrist biomechanics or carpal canal pressures during computer use.

#### Wrist position and exertion of force in computer work

Wrist positions and forces exerted by computer users have been measured in several studies. Keir et al[28], reported that wrist extension ranged from 23° to 30° and that ulnar deviation from -3.2° to 5.2° during mouse work. In a study of wrist position in keyboard work (entering of data), electrogoniometer measurements showed wrist extension of 14° and 20° at the 50^th ^and 90^th ^percentile, respectively [7]. In another study of keyboard work, mean ulnar deviation using a conventional keyboard was 18.9° (SD 6.8°) [29]. Gerr et al. reported wrist postures observed among 379 computer users. Mean wrist extension was 24.3° (SD 9.6°) during keyboard use and 23° (SD 8.8) during mouse use. Mean ulnar deviation was 5.0° (SD 7.3) during keyboard use and 1.0° (SD 7.7°) during mouse use [30].

Several investigators have measured finger tip forces among computer users. The finger tip force exerted while keying varied from less than 1 N to 7 N but in most studies was between 1 and 4 N [31-34].

#### Carpal tunnel pressure

In the literature search, 253 studies were retrieved and nine studies were found with measures of carpal tunnel pressure in relation to finger, wrist or arm use. Several studies have measured the carpal tunnel pressure (CTP) among persons free of CTS and among those with CTS [28,35-42]. In aggregate, these studies suggest that CTS development is associated with elevated CTP. The resting CTP with the wrist in neutral position among persons free of CTS ranges from 3 to 13 mmHg (results from 7 studies are summarized in [37] and [39]). CTP in CTS patients varies between 10 and 43 mmHg [39] though higher values have been found [38]. In an often cited study by Lundborg et al[35], CTP was increased experimentally among 16 human volunteers. In four participants the CTP was increased to 60 mmHg and in four other participants the CTP was increased to 90 mmHg. In these two groups, the sensory and subsequently the motor response were blocked within an hour. In a third group of 4 participants CTP was increased to 30 mmHg. This produced minor and varying effects but "pins and needles" was reported in 2 of 4 subjects. This was further studied by Gelberman et al. who found some functional loss at 40 mmHg and complete motor and sensory block at 50 mmHg among healthy subjects [36].

Several studies have measured the CTP profile associated with different wrist angles, finger flexion and forearm position [39,40,42,43]. The studies show that CTP is dependent on the position of the forearm, wrist and metacarpophalangeal joint (MCP). In particular, supination showed higher CTPs than pronation and MCP flexion increased CTP [28,39,42]. With wrist positions between 40° flexion and 40° extension the CTP did not exceed 20 mmHg regardless of MCP angle. Ulnar and radial deviation had only small effects [40].

CTP has been studied among persons engaged in actual work tasks. Rempel et al. measured CTP among 19 healthy subjects engaged in a number of hand intensive tasks [37]. CTP increased from 8 (SD 6) mmHg at rest to 18 (SD 13) mmHg after lifting 0.5 kilogram cans for 5 minutes at a rate of 20 cans per minute. Keir et al. conducted a study on the effect of computer tasks on CTP. Among 14 healthy subjects the mean CTP rose from 5.3 mmHg during rest to 16.8–18.7 mmHg (varying between different kinds of computer mice) with the hand static on the computer mouse and to 28.8–33.1 mmHg while dragging or pointing and clicking with the mouse [28]. This was the only study of computer work and CTP that was found.

To summarize, measurements of CTP under conditions commonly observed among computer users showed modest increases generally believed to be below potential harmful levels. However, one study showed an increase of CTP during actual mouse use to levels where possible neurological effects were seen experimentally. These studies have not been repeated in other studies and nothing is known about the effects of prolonged or repeatedly increased pressures to this level.

## Discussion

### The epidemiological evidence

The epidemiological evidence of an association between computer use and CTS is inconsistent. All 8 studies identified in this review that examined the association between computer work and CTS had important limitations. Thus, a definitive study that clarifies the relationship between computer use and CTS has not been conducted yet. Such a study should involve a large population with varying degrees of computer work, at least one year of follow-up, a careful exposure description and a precise CTS diagnostic procedure. Such a study would require considerable resources to complete.

Based on evaluation of study design, sample sizes and response rates, case definitions and the exposure information, the studies by Andersen et al., Thomsen et al. and Atroshi et al. were the most likely to yield valid inferences. In two of the studies very intense computer work was represented (e.g. data entry, graphical work) [7,8]. Andersen et al. observed an association between mouse use and symptoms of CTS in the median nerve distribution area in both the cross sectional and in the follow-up analyses. The association was statistically significant for participants reporting more than 20 h/w of mouse use with the risk almost tripled compared to the control group. A similar risk level was found in the study by Thomsen et al

Both studies had limitations. The study by Andersen et al. was performed during a time of intense debate on the potential hazards of mouse use in Denmark [8]. This may have influenced the results and thus explain why only associations with mouse use and not keyboard use was found. Information bias caused by beliefs about certain associations may have very strong effects. This was shown in a study of indoor climate symptoms where reporting turned out to be dependent on the information given to the participants about the purpose of the study [44] Another draw back of the study by Andersen et al. was the lack of NCT in the CTS case definition. Also of concern was the observation that associations with the most specific CTS case definition were not as strong as associations with the less specific CTS case definition.

Thomsen et al. used a sensitive and specific CTS case definition (including NCT) and precise estimates of exposure with the use of questionnaires and direct measurements. The odds ratio of 1.86 was based on only 8 cases among the exposed and no cases among the control group. Furthermore, the interpretation was complicated by the fact that participants with data entry exposure were pooled with participants performing manual letter sorting [7].

The study by Atroshi et al. showed quite convincingly the opposite of the expected, i.e. a negative association [14]. A limitation in this study could be the rather limited amount of keyboard work reported which would make it more difficult to show an effect. The exposure was self-reported and thus misclassification may have occurred. The possibility of reporting bias was limited because the participants were not aware of this special focus. The other cross-sectional study was difficult to interpret because of possible methodological bias, e.g. a much higher prevalence was found among men compared to women.

When statistical tests were applied to the results presented by Stevens et al., a statistically significant positive association was observed for the association between mouse use and CTS (although the authors concluded otherwise) [9]. However, the significant association was unadjusted for potentially confounding risk factors, e.g. age.

The case referent study by de Krom et al. was inconclusive. The number of exposed CTS cases in the study was very low and thus statistically unstable [10]. However, no pattern in the risk estimates was seen and all estimates were below unity. One of the strengths of the study was that the participants were blinded to the purpose of the study.

A number of methodological weaknesses substantially limited inferences that could be made form the study by Nathan et al. [11,12].

Findings in the longitudinal studies of repetitive low-force work pointed in different directions. Thus, these studies did not add further evidence.

The recent review by Palmer et al. reached the same overall conclusion concerning computer work based on only two epidemiological studies of the association [3]. Even though the present review managed to identify more studies of the association no further evidence was established mainly because of limitations in the studies.

This review also had limitations as well as strengths. The search strategy in the databases only identified six of the eight epidemiological studies. Therefore, we may have missed other studies with a focus on the use of keyboard, mouse or typing. Because of the relatively few studies in this field we preferred to describe the strengths and weaknesses of the studies in text in stead of using a scoring system as scoring systems are not always valid [45]. The risk of publication bias exists but is not obvious. There were both positive and negative studies among both the large and small studies.

### Pathophysiological mechanisms

Computer work involves very little force. Experiments on the effect of positions of fingers, wrist and forearm comparable to the positions common in computer use have shown that CTP increases but not to levels generally believed to be harmful [39,40,42]. Surprisingly, mean CTP levels between 28–33 mmHg where observed when study participants were dragging or clicking with the mouse. Lower values were found with the hand static on the mouse [28]. Although the experiment has never been repeated the findings indicate a possible pathophysiological mechanism for CTS among heavy mouse users.

## Conclusion

In summary, because of insufficient quality, bias, lack of consistency and statistical power evidence is insufficient to conclude that computer work (mouse and keyboard) causes CTS. As a consequence, this condition cannot be recognised as an occupational injury because of computer work. A large and unbiased prospective study is needed to establish further evidence. Such a study is recommended but the costs should be carefully considered.

## Abbreviations

CTS: carpal tunnel syndrome; NCT: nerve conduction test; CTP: carpal tunnel pressure; PPV: positive predictive value; NUDATA: Neck and Upper extremity Disorders Among Technical Assistants; PRIM: Project on Intervention and Research in Monotonous work; MCP: metacarpophalangeal.

## Competing interests

The authors declare that they have no competing interests.

## Authors' contributions

J.F. Thomsen was the lead author of the original document on which this manuscript was based and prepared this manuscript from the original document. F. Gerr and I. Atroshi made conceptual contributions to the original document and reviewed and edited this manuscript.

## Pre-publication history

The pre-publication history for this paper can be accessed here:



## Supplementary Material

Additional file 1 **Table 2.** Epidemiological studies on carpal tunnel syndrome and use of computer mouse and keyboard. The Table provides information on study design, population, response rate, control group, exposure, CTS case definition, confounders controlled for, results and strengths and weaknesses. Click here for file

## References

[B1] de Krom MC, Knipschild PG, Kester AD, Thijs CT, Boekkooi PF, Spaans F (1992). Carpal tunnel syndrome: prevalence in the general population. J Clin Epidemiol.

[B2] Atroshi I, Gummesson C, Johnsson R, Ornstein E, Ranstam J, Rosen I (1999). Prevalence of carpal tunnel syndrome in a general population. JAMA.

[B3] Palmer KT, Harris EC, Coggon D (2007). Carpal tunnel syndrome and its relation to occupation: a systematic literature review. Occup Med (Lond).

[B4] Thomsen JF (2005). Carpal tunnel syndrome and the use of computer mouse and keyboard. National Board of Industrial Injuries.

[B5] Jensen LK (2008). Knee osteoarthritis: influence of work involving heavy lifting, kneeling, climbing stairs or ladders, or kneeling/squatting combined with heavy lifting. Occup Environ Med.

[B6] Gerr F, Marcus M, Ensor C, Kleinbaum D, Cohen S, Edwards A (2002). A prospective study of computer users: I. Study design and incidence of musculoskeletal symptoms and disorders. Am J Ind Med.

[B7] Thomsen JF, Hansson GA, Mikkelsen S, Lauritzen M (2002). Carpal tunnel syndrome in repetitive work: a follow-up study. Am J Ind Med.

[B8] Andersen JH, Thomsen JF, Overgaard E, Lassen CF, Brandt LP, Vilstrup I (2003). Computer use and carpal tunnel syndrome: a 1-year follow-up study. JAMA.

[B9] Stevens JC, Witt JC, Smith BE, Weaver AL (2001). The frequency of carpal tunnel syndrome in computer users at a medical facility. Neurology.

[B10] de Krom MC, Kester AD, Knipschild PG, Spaans F (1990). Risk factors for carpal tunnel syndrome. Am J Epidemiol.

[B11] Nathan PA, Meadows KD, Doyle LS (1988). Occupation as a risk factor for impaired sensory conduction of the median nerve at the carpal tunnel. J Hand Surg Br.

[B12] Nathan PA, Meadows KD, Istvan JA (2002). Predictors of carpal tunnel syndrome: an 11-year study of industrial workers. J Hand Surg.

[B13] Ali KM, Sathiyasekaran BW (2006). Computer professionals and Carpal Tunnel Syndrome (CTS). Int J Occup Saf Ergon.

[B14] Atroshi I, Gummesson C, Ornstein E, Johnsson R, Ranstam J (2007). Carpal tunnel syndrome and keyboard use at work: a population-based study. Arthritis Rheum.

[B15] Palmer KT, Cooper C, Walker-Bone K, Syddall H, Coggon D (2001). Use of keyboards and symptoms in the neck and arm: evidence from a national survey. Occup Med (Lond).

[B16] Werner RA, Franzblau A, Albers JW, Buchele H, Armstrong TJ (1997). Use of Screening Nerve Conduction Studies for Predicting Future Carpal Tunnel Syndrome. Occupational and Environmental Medicine.

[B17] Werner RA, Gell N, Franzblau A, Armstrong TJ (2001). Prolonged median sensory latency as a predictor of future carpal tunnel syndrome. Muscle Nerve.

[B18] Gell N, Werner RA, Franzblau A, Ulin SS, Armstrong TJ (2005). A longitudinal study of industrial and clerical workers: incidence of carpal tunnel syndrome and assessment of risk factors. J Occup Rehabil.

[B19] Greening J, Lynn B (1998). Vibration sense in the upper limb in patients with repetitive strain injury and a group of at-risk office workers. Int Arch Occup Environ Health.

[B20] Greening J, Lynn B, Leary R (2003). Sensory and autonomic function in the hands of patients with non-specific arm pain (NSAP) and asymptomatic office workers. Pain.

[B21] Doezie AM, Freehill AK, Novak CB, Dale AM, Mackinnon SE (1997). Evaluation of cutaneous vibration thresholds in medical transcriptionists. J Hand Surg.

[B22] Overgaard E, Brandt LP, Ellemann K, Mikkelsen S, Andersen JH (2004). Tingling/numbness in the hands of computer users: neurophysiological findings from the NUDATA study. Int Arch Occup Environ Health.

[B23] Murata K, Araki S, Okajima F, Saito Y (1996). Subclinical impairment in the median nerve across the carpal tunnel among female VDT operators. Int Arch Occup Environ Health.

[B24] Jensen BR, Pilegaard M, Momsen A (2002). Vibrotactile sense and mechanical functional state of the arm and hand among computer users compared with a control group. Int Arch Occup Environ Health.

[B25] Sanden H, Edblom M, Ekman A, Tenenbaum A, Wallin BG, Hagberg M (2005). Normal nerve conduction velocity and vibrotactile perception thresholds in computer users. Int Arch Occup Environ Health.

[B26] Gerr F, Letz R, Harris Abbott D, Hopkins LC (1995). Sensitivity and specificity of vibrometry for detection of carpal tunnel syndrome. J Occup Environ Med.

[B27] Tanaka S, Mcglothlin JD (1993). A conceptual quantitative model for prevention of work-related carpal tunnel syndrome (CTS). International Journal of Industrial Ergonomics.

[B28] Keir PJ, Bach JM, Rempel D (1999). Effects of computer mouse design and task on carpal tunnel pressure. Ergonomics.

[B29] Marklin RW, Simoneau GC (2001). Effect of setup configurations of split computer keyboards on wrist angle. Phys Ther.

[B30] Gerr F, Marcus M, Ortiz D, White B, Jones W, Cohen S (2000). Computer users' postures and associations with workstation characteristics. AIHAJ.

[B31] Feuerstein M, Armstrong T, Hickey P, Lincoln A (1997). Computer keyboard force and upper extremity symptoms. J Occup Environ Med.

[B32] Rempel D, Dennerlein J, Mote CD, Armstrong T (1994). A method of measuring fingertip loading during keyboard use. J Biomech.

[B33] Smutz P, Serina E, Rempel D (1994). A system for evaluating the effect of keyboard design on force, posture, comfort, and productivity. Ergonomics.

[B34] Johnson PW, Hagberg M, Hjelm EW, Rempel D (2000). Measuring and characterizing force exposures during computer mouse use. Scand J Work Environ Health.

[B35] Lundborg G, Gelberman RH, Minteer-Convery M, Lee YF, Hargens AR (1982). Median nerve compression in the carpal tunnel – functional response to experimentally induced controlled pressure. J Hand Surg.

[B36] Gelberman RH, Szabo RM, Williamson RV, Hargens AR, Yaru NC, Minteer-Convery MA (1983). Tissue pressure threshold for peripheral nerve viability. Clin Orthop Relat Res.

[B37] Rempel D, Manojlovic R, Levinsohn DG, Bloom T, Gordon L (1994). The effect of wearing a flexible wrist splint on carpal tunnel pressure during repetitive hand activity. J Hand Surg Am.

[B38] Luchetti R, Schoenhuber R, Alfarano M, Deluca S, De Cicco G, Landi A (1994). Serial overnight recordings of intracarpal canal pressure in carpal tunnel syndrome patients with and without wrist splinting. J Hand Surg Br.

[B39] Weiss ND, Gordon L, Bloom T, So Y, Rempel DM (1995). Position of the wrist associated with the lowest carpal-tunnel pressure: implications for splint design. J Bone Joint Surg Am.

[B40] Werner R, Armstrong TJ, Bir C, Aylard MK (1997). Intracarpal canal pressures: the role of finger, hand, wrist and forearm position. Clin Biomech.

[B41] Keir PJ, Bach JM, Rempel DM (1998). Fingertip loading and carpal tunnel pressure: differences between a pinching and a pressing task. J Orthop Res.

[B42] Rempel D, Bach JM, Gordon L, So Y (1998). Effects of forearm pronation/supination on carpal tunnel pressure. J Hand Surg.

[B43] Keir PJ, Bach JM, Rempel D (1999). Effects of computer mouse design and task on carpal tunnel pressure. Ergonomics.

[B44] Brauer C, Mikkelsen S (2003). The context of a study influences the reporting of symptoms. Int Arch Occup Environ Health.

[B45] Moher D, Jadad AR, Tugwell P (1996). Assessing the quality of randomized controlled trials. Current issues and future directions. Int J Technol Assess Health Care.

