# Supplementary material for: Carpal tunnel syndrome and the use of computer mouse and keyboard: A systematic review
Source: BMC Musculoskelet Disord. 2008 Oct 6;9:134. doi: 10.1186/1471-2474-9-134 (PMC2569035; doi:10.1186/1471-2474-9-134)
Supplement: Additional file 1 — Table 2. Epidemiological studies on carpal tunnel syndrome and use of computer mouse and keyboard. The Table provides information on study design, population, response rate, control group, exposure, CTS case definition, confounders controlled for, results and strengths and weaknesses. [file 1471-2474-9-134-S1.doc]

Table 2 Epidemiological studies on carpal tunnel syndrome and use of computer mouse and keyboard. Only studies with symptoms confirmed with either nerve conduction test (NCT) or clinical interviews are included. The studies are listed chronologically according to publication year. RR=response rate, OR=odds ratio, PR=prevalence ratio, BMI=body mass index, h/d=hours/day, h/w=hours/week.

| **Study**  **Year** | **Design, population, response rate** | **Control group** | **Exposure measure** | **Case definition** | **Confounder control** | **Results** | **Strengths,**  **weaknesses** |
| --- | --- | --- | --- | --- | --- | --- | --- |
| Atroshi et al. 2007[14] | Population-based cross-sectional study.  2465 participants. RR (questionnaire) 82% (54% females). RR examinations 80% | 636 participants without computer work | Self reported daily hours with keyboard | Questionnaire symptoms verified in clinical examination and a positive NCT | Age, gender, height, hand temperature | Keyboard use >=1 h/d vs. <1 h/d:  PR 0.55 (0.32-0.96) | Population based. High response rates.  Self reported keyboard exposure could be biased by symptoms |
| Ali KM et al 2006[13] | Cross-sectional study. 648 (18% females) randomly selected from 4276 employees in 21 companies.  RR 100% | <4 years of computer work: 391  <8 hours of computer work/day: 84 | Years of computer work,  h/d of computer work | Pain, numbness median area past week at clinical interview and either positive Phalen’s or Tinel’s sign | Age, gender, smoking, alcohol use, BMI | 4-8 years: OR 2.1 (1.3-3.6)  >8 years:  OR 2.7 (1.3-5.8)  8-12 h/d:  OR 3.6 (1.3-10.3)  >12 h/d:  OR 4.4 (1.3-14.9) | Very long working hours.  No NCT, no blinding, self-report of exposure. Prevalence much higher among men (14.5% vs. 6.8%) |
| Andersen et al. 2003 [8] | 1 year follow-up study. 6943 technical assistants and machine technicians.  RR at baseline 73%, follow-up 60% of baseline eligible. (62% females). | 1279 / 532 within population with mouse /keyboard use <2.5 h/w | Self reported hours pr week with use of mouse and keyboard grouped in 5 hour groups | Two case definitions (questionnaire and interview): 1. Tingling, numbness at least weekly defined to median nerve area  2. As 1 including symptoms at night | Personal characteristics (including age, gender), psychosocial factors, physical work characteristics | Mouse use, baseline: Exposure-response pattern in def. 1. Def. 2 only significant >30h/w  Follow-up:  Significant risks above 20 h/w (def. 1) Keyboard:  No significant findings. | Separate risk estimates for mouse and keyboard time presented.  Indication of information bias because of the different results for mouse and keyboard use. |
| Gerr et al. 2002 [6] | 1.7 years follow-up study. 632 (71% females) newly hired. | 228 within population with <2 years of previous computer use | Self reported previous computer use | Questionnaire symptoms and NCT | Prior symptoms, personal characteristics (10 variables) | 3 prevalent CTS cases and 3 incident cases. | The effect of present computer work could not be assessed because of  too few persons with CTS. |
| Thomsen et al. 2002 [7] | 1.5 years follow-up study. 731 participants (74% females). 3 companies. RR at baseline 68-74%  Mixed exposure: Data entry work 59% of repetitive work hours | 219 within population with varied work | Company walk through. Self reported hours per week with job tasks. Goniometer measurements. | Questionnaire and interview symptoms, NCT. | Age, gender, seniority, BMI, forceful work | Baseline: Increased risk OR=1.86 (95% CI 1.06-3.19) for every 10 hour increase in repetitive work  Follow-up: Too few CTS cases | Few incident cases.  Mixed repetitive exposure though data entry was dominant. |
| Nathan et al. 1988, 2002 [11,12] | 11-year follow-up study. 471 participants at baseline (RR not reported), 256 (54%) at follow-up. 5 exposure groups.  22 keyboard operators in one group defined as light resistance, very high repetition | Administra-tive clerical work | Observation of job functions. | At baseline: Abnormal NCT alone. Prevalence 39%.  At follow-up: Abnormal NCT and CTS symptoms or reported CTS surgery.  Annual incidence 1.2%. | Baseline: Age.  Follow-up: 6 personal characteristics determined at baseline (but not age, gender). | Follow-up: Keyboard use OR 0.88 (95% CI 0.52-1.47). | Only 22 keyboard operators at baseline. No information about job changes in follow-up period.  Case definition at baseline based on NCT alone |
| Stevens et al. 2001 [9] | A case-referent study within a cross sectional design.  256 medical secretaries, RR 82% identified as frequent computer users.: Cases: 27 with CTS symptoms. | Participants without CTS symptoms  N=222 | Self reported h/d of keyboard use. Years of keyboard use.  No, occasional or frequent mouse use | Questionnaire symptoms combined with clinical interview confirming symptoms in median area. Prevalence 10.5%. | No adjustment (97% females) | Frequent mouse use: 48.1% and 27.9%, respectively (p=0.04). | The exposure gradient for keyboard use was small. Despite study conclusion a statistically significant difference in mouse use was found. |
| de Krom et al. 1990  [10] | Case-referent study.  156 cases, 28 recruited from population, 128 consecutively from clinic RR=70%  (84% females) | 473 controls from general population (RR=71%, 66% females) | Weekly hours of typing in 4 groups (0, 1-7, 8-19, 20-40). Information obtained by interview. | Questionnaire and interview symptoms, NCT. Prevalence in population sample 5.6%. | Gender, age and the interaction term gender*age. | Relative risk below 1 for all groups | Participants were blinded about the main focus.  Few “typing” cases (n=12) |
